# False-negative HIV tests using oral fluid tests in children taking antiretroviral therapy from Harare, Zimbabwe

**DOI:** 10.7448/IAS.20.7.21751

**Published:** 2017-08-29

**Authors:** Ioana D. Olaru, Grace McHugh, Suba Dakshina, Edith Majonga, Ethel Dauya, Tsitsi Bandason, Katharina Kranzer, Hilda Mujuru, Rashida A. Ferrand

**Affiliations:** ^a^ Division of Clinical Infectious Diseases, Research Center Borstel, Borstel, Germany; ^b^ Biomedical Research and Training Institute, Harare, Zimbabwe; ^c^ Department of Clinical Research, London School of Hygiene and Tropical Medicine, London, UK; ^d^ Department of Paediatrics, University of Zimbabwe, Harare, Zimbabwe

**Keywords:** HIV, misdiagnosis, rapid diagnostic test, oral fluid test, children

## Abstract

**Introduction**: Rapid diagnostic tests (RDT) for HIV infection have high sensitivity and specificity, but in the setting of longstanding antiretroviral therapy (ART), can give false results that can lead to misinterpretation, confusion and inadequate management. The objective of this study was to evaluate the proportion of falsely negative results of a RDT performed on oral fluid in HIV-infected children on longstanding ART.

**Methods**: One hundred and twenty-nine children with known HIV infection and receiving ART were recruited from the HIV Clinic at the Harare Central Hospital, Zimbabwe. HIV testing was performed on oral fluid and on finger-stick blood.

**Results and Discussion**: Children included in the study had a median age of 12 years (IQR 10–14) and 67 (51.9%) were female. Median age at HIV diagnosis was 5 years (IQR 3–6) and the median time on ART was 6.3 years (IQR 4.3–8.1). The oral fluid test was negative in 11 (8.5%) patients and indeterminate in 2 (1.6%). Finger-stick blood test was negative in 1 patient. Patients with a negative oral fluid test had a higher CD4 cell count (967 vs. 723 cells/mm^3^, *p* = 0.016) and a longer time on ART (8.5 vs. 6 years, *p* = 0.016).

**Conclusions**: This study found that a substantial proportion of false-negative HIV test results in children on longstanding ART when using an oral fluid test. This could lead to misinterpretation of HIV test results and in the false perception of cure or delayed diagnosis.

## Introduction

Rapid diagnostic tests (RDT) for HIV infection using whole-blood specimens have being used globally since 2005 [1]. These RDTs have high sensitivity, are easy to perform, require little or no infrastructure, and have a relatively low cost and a rapid turn-around time making them optimal for low-resource, high HIV burden settings. However, as with any test, the performance of the test will depend on its inherent sensitivity and specificity and the prevalence of the condition being tested for. The problem of false-positive test results especially in the context of low HIV prevalence is well recognized. Serial testing with a highly sensitive test followed by a confirmatory test with high specificity addresses this issue [2].

Although RDTs have been widely used both in health facilities and in community-based HIV testing and counselling approaches, a key barrier remains the reliance of a client making contact with a provider and receiving the test result from the provider, who may be known to the client. In recent years, there has been increasing interest in promoting self-testing as a strategy to address these barriers. Self-testing would enable individuals to undergo HIV testing confidentially and without concern about unwanted disclosure of their status to others. A recent meta-analysis of studies including adults at risk for HIV infection showed that HIV RDTs performed on blood had sensitivities and specificities exceeding 98–99% [3]. Oral fluid tests (OFTs) are RDTs that detect salivary HIV antibodies, and have been shown to have comparable performance to blood-based RDTs. As with blood-based RDTs, a positive OFT result can be confirmed by a subsequent blood-based test. In 2012, the first OFT received approval by the Food and Drug Administration as a home-use HIV kit for self-testing. The use of an OFT as a self-testing strategy has been demonstrated to be highly acceptable and accurate in Africa [4,5].

OFTs are particularly attractive for use in children because of their non-invasiveness. Studies have demonstrated a slow but persistent loss of HIV-specific antibodies in highly suppressed HIV-infected children and adolescents that may lead to false-negative results in blood-based RDTs [6]. HIV antibody titres in saliva are lower than antibody titres in blood, which may make OFTs more prone to false-negative results [3]. This appears to be more frequently encountered in the setting of longstanding ART and in individuals receiving pre-exposure prophylaxis (PrEP) [7,8]. We recently observed several cases of false negative HIV tests using OFT among children and adolescents taking antiretroviral therapy. Although this has already been described to occur in adults, there are no studies focusing on the paediatric population [9]. To further investigate this, we systematically evaluated the performance of the OFT compared to the blood-based RDT among perinatally HIV-infected children aged 7–18 years established on ART.

## Methods

The study was conducted in 2016 and was nested within an ongoing clinical cohort study among perinatally HIV-infected children on ART. Children with HIV who had been receiving ART for at least 18 months were recruited from the HIV Clinic at the Harare Central Hospital, Zimbabwe. HIV testing was performed using Ora-Quick ADVANCE HIV I/II™ OFT (OraSure Technologies, Bethlehem, USA) for oral fluid and concurrently using a finger-prick whole-blood sample (Alere Determine HIV 1/2, Alere Technologies, Jena, Germany). Testing was performed as per the instructions of the manufacturer by trained nurses. The nurse who performed the test was blinded to the result of the other test. CD4 count was assessed using the Alere PIMA CD4 analyser, and viral load was measured using GeneXpert HIV-1 Viral Load (Cepheid, Sunnyvale, CA). Demographic details, age at ART initiation and duration of ART use were collected.

Statistical analysis was performed using STATA version 14 (Stata-Corp, TX, USA). The Mann–Whitney U-test and Student’s t-test were used to evaluate for differences between groups for continuous variables. For categorical variables, the χ2 test was used. Multivariable logistic regression was used to examine for factors associated with a false negative OFT. The level of significance was set at α = 0.05.

Ethical approval for the parent study was obtained from the Medical Research Council of Zimbabwe, the Biomedical Research and Training Institute Institutional Review Board and the London School of Hygiene and Tropical Medicine Ethics Committee. Written informed consent from guardians and assent from participants were obtained. Specific verbal consent was also obtained to perform OFTs and finger-prick samples.

## Results and discussion

In total 129 participants were enrolled, with median age 12 years (IQR 10–14), and 67 (51.9%) being female (Table 1). The study participants had been diagnosed with HIV infection at a median age of 5 years (IQR 3–6) and the median duration on ART was 6.3 years (IQR 4.3–8.1). At the time of the OFT, the median CD4 cell count was 747 cells/mm^3^ (IQR 474–989) and 30 (34.9%) had a viral load exceeding 1000 copies/ml. The OFT was negative in 11 (8.5%) patients and indeterminate in two (1.6%). Finger-prick blood tests were negative in one patient (0.8%) who also had a negative OFT. Patients with a negative OFT had a higher CD4 cell count (967 vs. 723 cells/mm^3^, *p* = 0.016), a longer time on ART (8.5 vs. 6 years, *p* = 0.018) and were more likely to be girls (76.9% vs. 49.1%, *p* = 0.057). Furthermore, children with a negative OFT had a median age at ART initiation of 4.5 years, while those with a positive test had a median age of 6.2 years although this was not statistically significant (*p* = 0.138). Only 5 (3.9%) children were started on ART within their first year of life. There was no association between age at ART initiation and a false-negative OFT result. While this was a pre-defined variable to be included in multivariable analysis, this was not done due to a strong collinearity with duration of ART. Notably, 64% of those with a positive OFT had a viral load <1000copies/ml compared to 78% of those with a false-negative OFT result.

**Table 1.** Characteristics of patients by oral mucosal test result

	Total *n* = 129	Positive OMT *n* = 116	Negative or indeterminate OMT *n* = 13	*p*-Value
Female, *n* (%)	67 (51.9)	57 (49.1)	10 (76.9)	0.057
Age at study visit (years), median (IQR)	12 (10–14)	12 (10–14)	12 (11–15)	0.464
Age at HIV diagnosis (years), median (IQR)	5 (3–6)	5 (3–6)	4 (1–7)	0.415
Age at ART initiation (years), median (IQR)	6 (4–8.3)	6.2 (4.1–8.4)	4.5 (1.4–7)	0.138
Time on ART (years), median (IQR)	6.3 (4.3–8.1)	6 (4.1–8)	8.5 (7.2–9.4)	0.018
Current CD4 cell count (cells/µl), median (IQR)	747 (474–989)	723 (457–928)	967 (754–1414)	0.016
*Viral load <1000 copies/ml, *n* (%)	56 (65.1)	49 (63.6)	7 (77.8)	0.400

*data available for 86 participants only. Of the total number of individuals where the viral load measurement was available, 9 had a negative or indeterminate OMT and 77 had a positive OMT.

This study shows that a substantial proportion of children and adolescents receiving ART have a false-negative or indeterminate HIV test result using an OFT. Significantly more false-negative results occurred using an OFT compared to a whole-blood-based HIV RDT. While false-negative RDT results, can be due to technical issues such as inappropriate performance and self-interpretation of the test, this was not the case in this study where HIV testing was performed by trained nurses certified to provide HIV testing, and the oral fluid and the blood-based RDTs were performed concurrently [10].

False-negative test results on blood-based antibody tests have been shown to occur very early or very late in the course of disease [11], as well as a slow loss of HIV-specific antibodies among children with longstanding ART [6]. In addition, false-negative HIV tests have been reported in infants started on ART therapy soon after birth who were HIV DNA PCR-positive [12,13]. This may be explained by the decreased antigen presentation due to longstanding suppressed viral replication. Similarly, it could also be associated with the time between infection and ART initiation. For example, false-negative tests have been reported in children with perinatal HIV infection who were started on ART within the first months of life [14]. Furthermore, PrEP was shown to be associated with a delayed time to development of a reactive OFT when compared to placebo [15]. Since there appears to be an association between the early initiation of ART and test performance, false-negative OFTs while on treatment may become more common in both paediatric and adult populations due to the global move towards immediate treatment initiation following a positive HIV test. This underscores the importance of patient counselling to understand the implications of HIV infection and therapeutic goals for ART.

The sensitivity of the oral fluid-RDTs is high, reaching up to 100% (95%CI 97.9–100) when used for HIV screening of individuals who have never received ART [16]. However, this does drop among those who are taking ART. A longer duration of ART use and a high CD4 cell count were independently associated with a false-negative OFT in our study (Table 2). Those with a suppressed viral load appeared more likely to have a false-negative test, although we were not able to formally test for this association due to the large proportion of participants on whom viral load data was unavailable. Taken together, these findings imply that in this age group, those who have been on longstanding ART and robust immunological status have too low levels of antibodies to be detectable by OFTs. Although not statistically significant, an interesting finding was the higher rates of false-negative OFT test in females, although there was no association of gender with false-negative tests.

**Table 2.** Factors associated with false-negative or indeterminate oral fluid-based HIV test

Variable	OR (95% CI)	aOR (95% CI)
Female	4.66 (0.96; 22.47)	4.21 (0.81; 21.89)
Duration of ART (years)	1.30 (1.03; 1.64)	1.31 (1.01, 1.69)
CD4 count >750 cells/µl	10.00 (1.24; 80.61)	9.50 (1.13; 79.62)

Oral fluid-based RDTs are an attractive test for self-testing, as it is convenient to use and ensures anonymity and confidentiality [17]. In some settings, oral fluid-based tests are available over-the-counter or through online purchase In addition, the World Health Organization is encouraging countries to initiate pilot projects to implement and evaluate effective strategies for HIV self-testing as a means of achieving universal coverage of HIV testing [18]. Belief in faith healing or in the use of alternative treatments to cure HIV has been commonly reported in some populations with individuals living with HIV undergoing retesting to check for cure [19]. In a study in Tanzania, 44% of participants to a study believed that certain alternative treatments can cure HIV [20], and in another study seeking cure at a faith healer was associated with a significant decrease in treatment adherence [21]. In the absence of adequate counselling and patient education, a false-negative test result may lead to a wrong perception of cure, leading to ART interruption and exit from HIV care [12]. Additionally, with the scale up of PrEP, there is a possible risk of delayed HIV diagnosis given the longer time required for OFT to become positive in individuals taking PrEP. Furthermore, false-negative tests might cause the underestimation of HIV prevalence in surveys if participants under-report their HIV status. In a recent survey we conducted among 7–18 year olds, 12.9% of HIV-infected participants had a false-negative oral-fluid-based HIV test result *(manuscript in preparation).*

The limitation of this study is that it included a relatively small number of children from one centre and the lack of a longitudinal assessment. In addition, viral load tests were missing in a third of patients and therefore the association between viral load suppression and false-negative OFT test results could not be reliably examined.

In conclusion, 10% of older children and adolescents with HIV infection who were on longstanding ART had falsely negative or indeterminate HIV test results when using the oral fluid HIV test. Awareness of the possibility of false-negative results among healthcare providers and patients taking ART as well as among clients accessing PREP is critical, as self-testing is scaled up. Clear counselling and appropriate messaging are important to avoid misinterpretation of HIV test results, which could result in the false perception of cure or delayed diagnosis of HIV infection among those accessing PREP. Additionally, improving sensitivity of OFTs, counselling to prevent their use in individuals already diagnosed with HIV infection and new testing strategies are of paramount importance to avoid confusion and misunderstanding.
